# Retraction of “circRNA_0001679/miR-338-3p/DUSP16 axis aggravates acute lung injury”

**DOI:** 10.1515/med-2023-0891

**Published:** 2023-12-31

**Authors:** Jiang Zhu, Fukuan Zhong, Futao Chen, Yang Yang, Yingying Liao, Lifeng Cao, Yong Zhou, Qiaohong Bai

**Affiliations:** Department of Respiratory, The Second People’s Hospital of Lianyungang, Lianyungang 222023, Jiangsu, China; Department of Respiratory, The Second Hospital of Nanjing, Nanjing University of Chinese Medicine, Zhongfu Road 1, Gulou District, Nanjing 210003, Jiangsu, China

Retraction of: Zhu J, Zhong F, Chen F, Yang Y, Liao Y, Cao L, Zhou Y, Bai Q. circRNA_0001679/miR-338-3p/DUSP16 axis aggravates acute lung injury. Open Medicine 2022;17(1):403–413, DOI: 10.1515/med-2022-0417.

This article has been retracted by the publisher due to irregularities in the figures and lack of clarity regarding the origin of the images. As a result, the editors cannot guarantee the legitimacy of the presented data.

